# The alliance formation puzzle in contests with capacity-constraints: A test using American football reception-coverage contest data

**DOI:** 10.1371/journal.pone.0227750

**Published:** 2020-03-04

**Authors:** Justin Ehrlich, Matthew Harmon, Shane Sanders

**Affiliations:** 1 Syracuse University, Syracuse, NY, United States of America; 2 NFL.com, NFL, Syracuse, NY, United States of America; Baylor University, UNITED STATES

## Abstract

We utilize a contest-theoretic model to demonstrate a version of the alliance formation puzzle that aligns with reception-coverage contests in American football. Namely, secondary defenders can opt for single-coverage—1 v 1 contest. Alternatively, they can choose to ally—form double-coverage or 2 v 1 contest with exogenous intra-alliance prize division—when defending a given receiver. In our theoretical treatment, we find that defenses have a lower equilibrium success rate in preventing the receiver from “getting open” under double-coverage than under single-coverage in the absence of capacity constraints. We also find that this success rate paradox is a necessary condition for the alliance formation puzzle. We then test the theoretical treatment by analyzing 8,508 plays of NCAA and NFL game data within a set of fixed effects, logistic regression models that control for receiver, level-of-play, and season-of-play. We find that equilibrium level of defensive success rises significantly and substantially (p-value < 0.01 and marginal effect of between 13 and 17 percentage points) when moving from single-coverage to double-coverage, *ceteris paribus*. There is strong evidence that the necessary condition for the alliance formation puzzle does not hold in this setting. We conclude that sufficiently-binding physiological and training-based capacity constraints eliminate the alliance formation puzzle in this setting, as was shown theoretically by Konrad and Kovenock (2009). This empirical result suggests that other contest settings that regularly feature alliance, such as liquidity-constrained conflict, may not be puzzling.

## I. Introduction

Within a standard, three-party contest setting, alliance formation can be puzzling. Skaperdas [1] shows that two-party alliance formation within three-party contest decreases expected payoff of allied parties. Konrad [2], Kovenock and Roberson [3], Konrad and Kovenock [4], and Ke, Konrad, and Morath [5] further document conditions and settings under which the puzzle emerges. Moreover, Ke, Konrad, and Morath [5] find experimental evidence of puzzling alliance formation in contest, and Beekman, Cheung, and Levely [6] explore conditions for cooperation within contest environments. Assuming exogenous prize-sharing between allies, the puzzle emerges from a bi-lateral free-ridership problem among allies, whereby a party’s marginal benefit of input allocation decreases in number of allied arms allocated (see, e.g., [7–12]). This problem causes an under-allocation of arms by an alliance that, in turn, creates a loss of expected payoff and of victory likelihood among allies.

In an important theoretical work, Konrad and Kovenock [4] find that capacity constraints can explain alliance formation in contest. If parties to contest face input-allocational constraints, then the free-ridership problem does not necessarily doom a two-party alliance in three-party contest. Herein, we empirically test for presence of an alliance formation puzzle in a real-world contest setting that features capacity constraints and in which three-party contest frequently emerges. Namely, we test whether alliance formation is puzzling within reception-coverage contest trials between American football receivers and alliances of American football secondary defenders. American football players face capacity constraints in the sense that there is a limited amount of speed, agility, and effort that each can put forth in a given trial. Speed and effort, for example, are largely viewed as endowments or, at the very least, not subject to spot improvement within the course of a game. Despite the insistence of athletes and coaches that 110 percent effort is possible—that the effort ceiling is variable for a given player—one’s effort ceiling for a given play is well-defined and governed by physical and physiological constraints.

**Fig 1:**
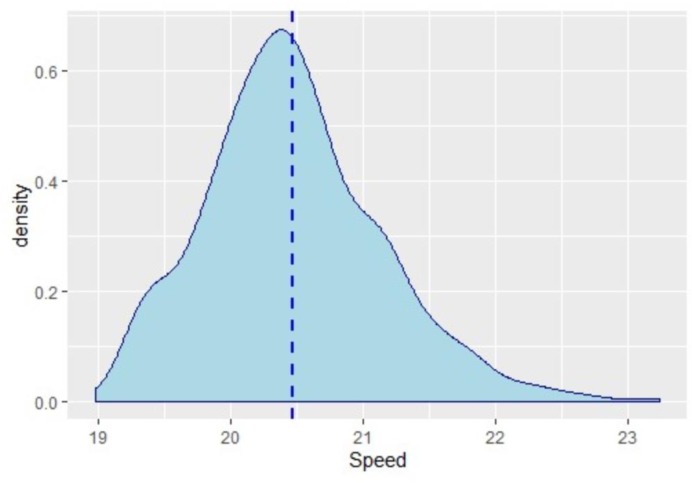
Density plot of NFL receiver maximum route speeds.

Given recent advances in player tracking technology, we can obtain salient measurements of player effort. From this, there is some evidence that NFL players perform at or near capacity within games. While it remains difficult to measure the efforts of receivers and defenders, one salient effort proxy for receivers is maximum in-game route speed. The following figure represents a density plot of maximum speed during given routes for receivers in the NFL from 2016–2018. The data was scraped from nextgenstats.nfl.com and represents all available receiver route speed data since the variable was tracked on the site. To the authors’ knowledge, this is the only source of instantaneous, in-game receiver speed data. There is no such data available with respect to defenders who cover receivers. Fig 1 is presented as follows.

The dashed, vertical line on the plot represents the average, estimated maximum speed of receivers at the NFL Draft Combine 40 Yard Dash. Though maximum speed is not tracked during the Combine, this average was estimated using a difference of splits [13]. Unfortunately, there is insufficient NFL Draft Combine data to obtain a *distribution* of maximum speeds for the 40 Yard Dash. It is important to note that the NFL Draft Combine 40 Yard Dash is run without pads or helmet and is often run with lightweight cleats. Despite the extra equipment weight that players carry during games, we take from the figure that receivers regularly reach in-game route speeds. This is the case also in spite of the fact that many receiver routes are not conducive to the achievement of maximum speed. That is, receivers do not often run purely straight line routes. In the absence of defender player tracking data, we assume that there is nothing peculiar about receivers and that secondary defenders exhibit similar effort characteristics.

By comparing receiver success rates conditional on being single-covered and double-covered, respectively, we are able to test for a primary feature of the alliance formation puzzle within a real-world contest (i.e., that the formation of an alliance lowers allied success rate). As the real-world setting of interest features capacity constraints among contestants, moreover, we can specifically test a central aspect of [4] capacity constraints solution to the alliance formation puzzle.

The remainder of the paper proceeds as follows. Section 2 lays out the standard contest theory of two-party alliance formation in three-party contest. In previous literature, this 2 v 1 setting is compared to three-party contest without alliance formation (i.e., 1 v 1 v 1 or three parties simultaneously fighting one another). There is no analogy to the 1 v 1 v 1 setting within American football, as there are only two teams represented in a given game. Therefore, we will compare 2 v 1 contest to a 1 v 1 or single-coverage reference case. We demonstrate in Section 2 that the alliance formation puzzle remains absent capacity constraints as a theoretical result given a 1 v 1 reference case. That is, a standard theoretical treatment absent capacity constraints yields that the defense enjoys a lower success rate and a lower payoff in the 2 v 1 setting than in the 1 v 1 setting. If our empirical results are consistent with these theoretical results, then a necessary condition for the alliance formation puzzle is met within this empirical setting. Such an outcome would raise questions as to why safeties are designated to double-coverage if such an action is counterproductive to team and player goals. If our empirical results are inconsistent with these theoretical results, then a necessary condition for the alliance formation puzzle is not met within this empirical setting. Under this outcome, we conclude that there is no alliance formation puzzle in the empirical setting of interest. Rather, player effort capacity constraints erase the alliance formation puzzle and explain alliance formation in NFL reception-coverage contest trials.

Section 3 describes the novel reception-coverage data that we utilize in the present study. Section 4 analyzes the data and discusses results, and Section 5 concludes.

## II. Alliance formation in a tullock contest with capacity constraints

In American football reception coverage, defensive players can form an alliance to collectively defend against a receiver. The purest such alliance is a double-coverage scenario in which the cornerback (C) and safety (S) defend the same receiver (R) of the opposing team. Such a scenario can be modelled as a three-party contest trial, in which C and S ally against R to prevent R from achieving separation (i.e., from fleeing coverage). This three-party scenario can be compared to another common coverage scheme in which C single-covers R. The single-coverage scenario can be modelled as a two-party contest trial between C and R.

The level of effort by player i is denoted as e_i_, where i ∈ {C,S,R}. In each contest setting, the value of victory to each player is represented by M_i_, where M_i_ can be thought of as the expected present value of future monetary rewards to player i from victory on a given contest trial [14]. However, there is tremendous growth potential from these averages [15], with an average salary of roughly $40,000-$50,000 per snap [16]. However, top players at these positions can earn roughly $250,000-$500,000 per snap. As such, average players can earn a potentially large return on performance with marginal performance improvements. From another perspective, there is high turnover within NFL skill positions such that an average cornerback may be out of the League on the basis of his performance over a fairly small number of plays. From either perspective, it is reasonable to expect a typical value of M_i_ for a contest trial to be in the thousands of dollars (or perhaps in the tens of thousands of dollars given a high-leverage game situation).

For analytical tractability, we assume that the sum of rewards to each side of the contest is equal and normalized to one. That is, M_R_ = 1 and M_c_ + M_S_ = 1 under three-party contest with alliance, whereas M_R_ = 1 and M_c_ = 1 under two-party contest. Party C, the coverage specialist, will either defend R alone or form an alliance with Party S, the defensive secondary generalist, to double-cover R. We do not consider zones or presses but, rather, only single or double coverage. Zones and presses are defensive schemes under which loose coalitions can form and dissipate. Parties C and S share in the payoff in the event that they collectively contain the receiver. When C single-handedly contains R, however, he receives the same payoff of 1 unit privately. This payoff structure is consistent with that of Konrad and Kovenock [4], Ke, Konrad, and Morath [5], and others.

### Double-coverage reception contest

Under the double-coverage scenario, we take the two defenders as sharing equally in the contest reward. Indeed, it is typically difficult to assign unequal credit under true double-coverage. Even if one defensive player is beaten (e.g., by “playing under” R), the beaten player’s actions help his defensive teammate make the right play (e.g., to play “over the top” without worrying about being beaten “under” R). Despite this award-splitting arrangement, the two defenders have the discretion to expend their efforts independently.

Under the double-coverage arrangement, the probability of success for the coalition defense, denoted as P_CS_, and that for the receiver are:

$$P_{CS}=\frac{{e_{C}+e}_{S}}{{e_{C}+e}_{S}+e_{R}}\;and\;P_{R}=\frac{e_{R}}{{e_{C}+e}_{S}+e_{R}}$$

The expected payoffs of the three players given equal prize division within alliance are then specified as

$$E(\pi_{C})=\frac{{e_{C}+e}_{S}}{{e_{C}+e}_{S}+e_{R}}∙\frac{1}{2}-e_{c}$$ (1A)

$$E(\pi_{S})=\frac{{e_{C}+e}_{S}}{{e_{C}+e}_{S}+e_{R}}∙\frac{1}{2}-e_{S}$$ (1B)

$$E(\pi_{R})=\frac{e_{R}}{{e_{C}+e}_{S}+e_{R}}∙1-e_{R}$$ (1C)

In a simultaneous-move game, we have from (1a) to (1c) that the first-order conditions for the expected payoff maximization are:

$$\frac{e_{R}}{{{(e}_{C}+e_{S}+e_{R})}^{2}}∙\frac{1}{2}=1$$ (2A)

$$\frac{e_{R}}{{{(e}_{C}+e_{S}+e_{R})}^{2}}∙\frac{1}{2}=1$$ (2B)

$$\frac{e_{C}+e_{S}}{{{(e}_{C}+e_{S}+e_{R})}^{2}}=1$$ (2C)

Solving Eqs (2A)–(2C), we have the Nash equilibrium levels of effort by the coalition defense and the receiver as follows:

$${{(e}_{C}+e_{S})}^{*}=\frac{1}{9}\;and\;e_{R}^{*}=\frac{2}{9}$$

Given that allied first order conditions are identical, the system of first order conditions is underdetermined such that $e_{C}^{*}$ and $e_{S}^{*}$ cannot be solved for uniquely. However, (e_C_ + e_S_)* and $e_{R}^{*}$ can be solved for uniquely. It follows that the probability of success in the contest for the coalition defense and that for the receiver are:

$$P_{CS}^{*}=\frac{1}{3}\;and\;P_{R}^{*}=\frac{2}{3}$$

The equilibrium levels of expected payoffs are calculated as

$${\left(E(\pi_{C})+E(\pi_{S})\right)}^{*}=\frac{2}{9}\;and\;E{\left(\pi_{R}\right)}^{*}=\frac{4}{9}$$

### Single-coverage reception contest

We next consider a two-player contest between the cornerback and receiver (i.e., a single-coverage treatment). As this is nothing more than a standard, two-player Tullock [17] contest, we can summarize the equilibrium payoffs and outcomes as follows:

$$e_{C}^{*}=e_{R}^{*}=\frac{2}{9}$$

$$E{{\left(\pi_{C}\right)}^{*}}^{\prime }=E{{\left(\pi_{R}\right)}^{*}}^{\prime }=\frac{1}{4}$$

$${P_{C}^{*}}^{\prime }={P_{R}^{*}}^{\prime }=\frac{1}{2}$$

**Theoretical result.** Holding player ability levels constant, we find that the defense has a lower effort allocation, equilibrium success rate, and expected payoff in single-coverage (i.e., 1 v 1) than in double-coverage (i.e., 2 v 1) in the absence of capacity constraints.

In its present form, with exogenous within-alliance prize division, the alliance formation puzzle arises solely from a free ridership problem. In an alternative setting of endogenous within-alliance prize division, iterated contest also contributes to the alliance formation puzzle. In and of itself, free-ridership is not a problem to allied parties. It decreases the cost of contest, which represents a welfare gain. However, this cost savings is unilateral. The unallied opponent arms as in the case of 1 v 1 v 1 contest, and the alliance’s likelihood of victory subsequently decreases. Hence, a lower likelihood of allied contest victory from free ridership is a necessary condition for the alliance formation puzzle under exogenous intra-alliance prize division. If an alliance formation puzzle is present in the sense that the sum of allied payoffs are lower, then it must be the case—again, under exogenous prize division—that allies have a lower likelihood of victory for having allied. Without this effect, free-ridership would generate only a cost savings to allies, and expected payoffs would rise. In our empirical test for the presence of an alliance formation puzzle, we focus upon the success rate aspect of the alliance formation puzzle, as play-level player payoffs are not typically salient or even estimable in American football. As the success rate paradox is a necessary element of the alliance formation puzzle within the empirical setting, success rate provides an accessible and clear indicator as to the presence of the alliance formation puzzle. In S1 Appendix, we provide a theoretical treatment that is similar to this primary treatment. However, defenders split any prize from containment endogenously within the Appendix treatment. The endogenous prize division model works as follows. Defenders work together in an attempt to contain the receiver in a 2-on-1 scenario. They ally to prevent the receiver from “gaining separation.” As such, the first stage is a 3-party game with defenders allied to stop the receiver. In the event that the defenders succeed in containing the receiver in the first stage, they then compete with one another in a second stage nested contest to establish better apparent position at the close of the play, where the winner of this second stage contest receives credit for the containment. This second stage nested contest between defenders often appears as a jockeying for position among defenders and even carries on after the play finishes in many cases, whereby each defender indicates that he in fact stopped the play by drawing an “X” with his arms. As stated, the S1 Appendix treatment allows for unequal credit and retains the result of the alliance formation puzzle. We provide this treatment to demonstrate the robustness of the alliance formation puzzle result.

Konrad and Kovenock [4] demonstrate that alliances improve the outcome of allied parties—both in terms of success likelihood and in terms of expected payoff—in the presence of *sufficient* binding capacity constraints. In the subsequent empirical section, we test whether the success of receivers in achieving separation from defender(s) is consistent with the presence of an alliance formation puzzle. That is, we test whether double-coverage is more or less successful than single-coverage in containing given sampled receivers. The empirical analysis utilizes sport data to test for the presence of a central and necessary aspect of the alliance formation puzzle within a real-world environment that features physiological and training-based capacity constraints.

## III. Data description and summary

Our study takes advantage of a novel data set constructed—purely for football analysis purposes at the time of its construction—by Harmon [18]. By watching hundreds of hours of game tape for the 2014 and 2015 NFL and 2015 NCAA seasons, Harmon was able to record—for more than 10,000 plays—the coverage scheme for each receiver on each play, the receiver’s identity, and whether the receiver was able to “get open” (flee or separate from coverage) at some point during the play by a sufficient margin to create a “reasonable target” for the quarterback [18]. While this reasonability criterion is somewhat subjective, Harmon utilized a systematic charting [18] and pause/slow-motion video features to remain as precise as possible in charting contest trials. Moreover, the data was rendered for the alternative purpose of rating top receivers or receiver prospects. For present purposes, this original purpose is convenient in the sense that the data is highly-relevant to the present study but was collected without any prior knowledge of the contest alliance formation puzzle; the primitive data collector was blind to the present research question at the time of recording.

The interaction between receivers and secondary defenders represents a self-contained contest within each play of a football game, whereby the receiver attempts to “get open,” and the secondary defender (defenders) attempts (attempt) to prevent such an occurrence. These contest trials are self-contained in the sense that the receiver’s ability to get open is not affected by such factors as quarterback decision-making or accuracy. In a true single or double-coverage scenario, moreover, the relevant secondary defender (defenders) is (are) designated to defend a particular receiver without discretion to assist in other defensive tasks. In this sense, single and double coverage are distinct from zone or press coverage, two types of coverage that were not considered herein. Zone and press coverage plays were not included in the data set, as they have no clear analogy in the alliance formation puzzle problem.

Harmon [18] collected data for more than 10,000 reception-coverage trials, 7,952 of which occurred single-coverage defensive scheme and 556 of which occurred under a double-coverage defensive scheme. This subset of 7,952 + 556 = 8,508 reception-coverage contest trials constitute the sample data for the present study. We summarize defensive coverage success by season/league and defensive scheme in Table 1.

**Table 1:** Defensive coverage success by Season/League and Defensive Scheme.

Season/League	|Receiver-seasons|	Defense Success Rate (Single-Cover)	Defense Success Rate (Double-Cover)
**2014 NFL**	25	1,191/3,270 = 0.364	135/280 = 0.482
**2015 NFL**	20	985/2,674 = 0.368	73/157 = 0.465
**2015 NCAA**	21	690/2,008 = 0.344	68/119 = 0.571
**Overall**	66	2,866/7,952 = 0.360	276/556 = 0.478

**Fig 2:**
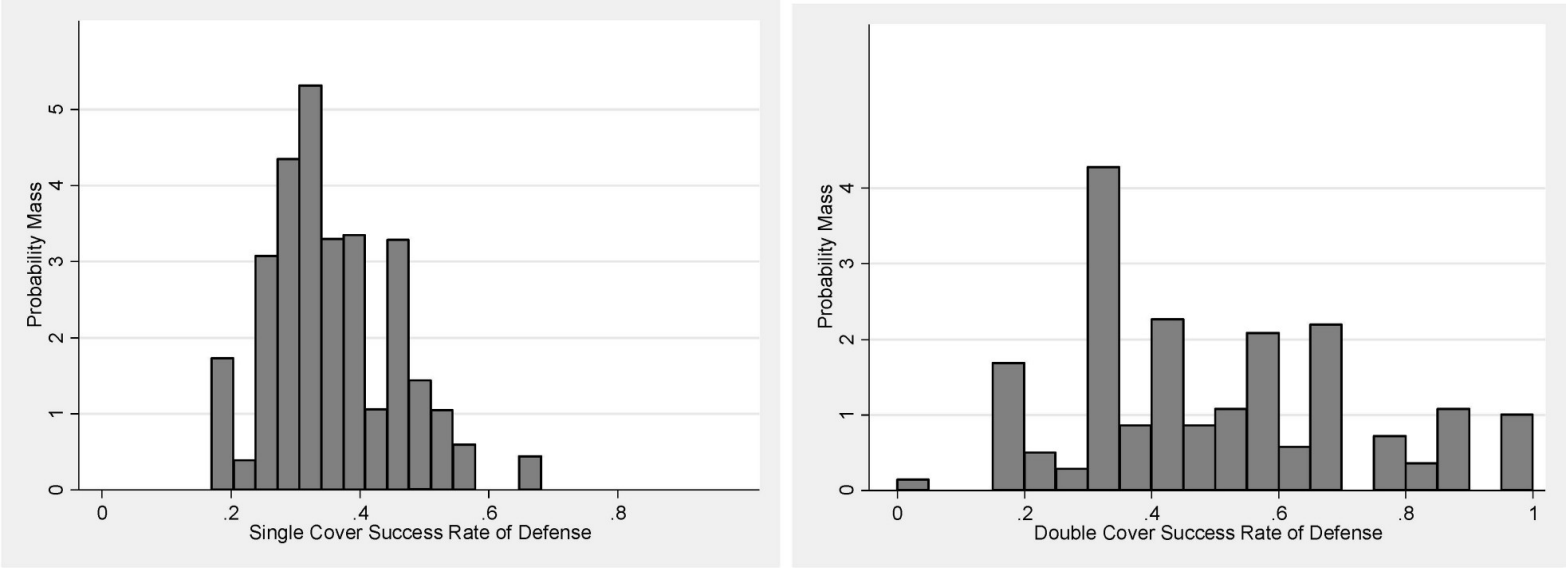
Mean defense coverage success rate by receiver-season (single cover (left); double cover (rt).

We note that double-coverage was experienced broadly among sampled receivers. Only one sampled receiver in the data did not draw any double-coverage. In the sample, we observe a higher defensive success rate under double-coverage. This elevated rate is most pronounced in the collegiate sub-sample but is present in each of the three sub-samples. Overall, the sampled defensive success rate is approximately 12 percentage points higher under double-coverage than under single-coverage. In Fig 2, we display a histogram of defensive coverage success rates by receiver defended.

These histograms are roughly analogous to sampling distributions in the sense that they represent a distribution of mean defensive coverage success rates across different sub-samples. However, the sub-samples are not taken from the same population but, rather, with distinct receivers being covered. One observes from these histograms that the defense is more successful, on average, under double-coverage, with approximately one-fifth of double-coverage success rates above 0.68: the maximum defensive success rate under single-coverage of a given receiver. However, defensive success rates vary to a greater degree across receivers under double-coverage than under single-coverage. This latter observation follows at least partly from a lower number of observations per receiver defended under double-coverage.

For each trial within the data, the unique receiver was identified, as was the level of play, NCAA or NFL, the season of play, 2014 or 2015, and the type of coverage. In total, samples from 66 receiver-seasons and 62 unique receivers are represented in the data set. All variables of interest, along with a representative *Receiver Fixed Effect* variable, are summarized in Table 2.

**Table 2:** Summary table for variables of interest.

Variable	Obs.	Mean	St. Dev.	Min	Max
Owin	8,508	0.631	0.483	0	1
Dwin	8,508	0.369	0.483	0	1
Singlecover	8,508	0.935	0.247	0	1
Doublecover	8,508	0.065	0.247	0	1
NFL_2014	8,508	0.417	0.493	0	1
NFL_2015	8,508	0.333	0.471	0	1
NCAA	8,508	0.250	0.433	0	1
receiverFE1	8,508	0.016	0.126	0	1

The variable *owin* (*dwin*) is a dummy variable that tracks whether the receiver (defensive secondary) “won” a play, *singlecover* (*doublecover*) is a binary variable that tracks whether the receiver was single-covered (double-covered) on a given play, and *NFL_2014* (*NFL_2015*, *NCAA*) equals 1 if the play occurred during the 2014 NFL season (2015 NFL season, 2014 NCAA season) and 0 otherwise. *ReceiverFE1* is the fixed effect binary variable for the first receiver. It is displayed to demonstrate a representative *Receiver Fixed Effect* variable. These summary statistics inform us that sampled receivers are able to gain separation on almost two-thirds of plays in which they are either single-covered or double-covered. We also find that single-coverage is employed much more often than is double-coverage. Single-coverage plays represent more than 93 percent of the data set. In the S2 Appendix tables at the close of the paper, we summarize defensive success rates against each receiver in the data set. In those tables, we also list receiver’s name, as well as season and level of play in which the receiver competed.

This dataset was originally collected for the purpose of rating top receivers. Therefore, receivers (reception-coverage trials) for the NCAA sub-data were included in the dataset based upon status as a top-projected receiver in the subsequent NFL Draft. For the NFL sub-data, receivers were included in the data based upon status as a top-drafted receiver in one of the two previous NFL Drafts. Among these prospects, only those receivers who played at least 300 snaps during the season and an average of at least 20 per game were included in the data. Given scarcity of resources and availability of game footage, Harmon obtained eight (six) games of footage for each identified NFL (NCAA) receiver in the season of interest. The sampled games for each sampled receiver-season represent the first eight (six) available games of footage for that player in that season. The data were first published on Harmon’s website [18] and generated a great deal of interest from the football analytics community. For the purpose of this paper, we have posted the formatted data [19], along with all paper code, to github and dataverse repositories available at *https*:*//github*.*com/Syracuse-University-Sport-Analytics/Alliance-Formation-Puzzle-in-Professional-Football* and *https*:*//dataverse*.*harvard*.*edu/dataset*.*xhtml*?*persistentId=doi%3A10*.*7910%2FDVN%2FZIQQXO&showIngestSuccess=true*, respectively.

Unfortunately, defenders were not identified for given reception-coverage trials within the data. This omission occurred because a) the primary purpose of the data was to rate receivers, b) receivers face a roughly similar distribution of defenders given a sufficiently large sample of plays (conditional on level of play), and c) the data recording process—already having taken several hundred hours—would have been greatly extended were defenders to have been identified. Using institutional facts about single and double-coverage, however, we will compensate for this omission when analyzing the fixed effects, logistic regression analysis.

## IV. Model and results

All of the following models and tests were conducted in Stata/SE 14.2 and is available with an open source license at online software repository [20]. The protocol of the methodology is documented at an open [21]. We consider a set of logistic regressions featuring receiver fixed or random effects or setting of play fixed effects. We cannot consider the two fixed effects simultaneously, as only 4 of the 62 receivers played in more than one setting (i.e., more than one of NCAA, 2014 NFL, and 2015 NFL). The four model specifications are given as follows.

$${dwin}_{i,j}=\beta_{0}+\beta_{1}doublecover_{i,j}+\beta_{2}{NFL}_{i}+e_{i,j}\;[Empirical\;Model\;1]$$

$${dwin}_{i,j}=\beta_{0}+\beta_{1}doublecover_{i,j}+\beta_{2}NFL14_{i}+\beta_{3}NFL15_{i}+e_{i,j}\;[Empirical\;Model\;2]$$

$${dwin}_{i,j}=\beta_{0}+\beta_{1}doublecover_{i,j}+\beta_{R}receiver_{i}+e_{i,j}\;[Empirical\;Model\;3]\;Random\;Effects$$

$${dwin}_{i,j}=\beta_{0}+\beta_{1}doublecover_{i,j}+\beta_{R}receiver_{i}+e_{i,j}\;[Empirical\;Model\;4]\;Fixed\;Effects$$

We note that a Hausman specification test supports use of fixed over random effects (Chi-sq test statistic value = 8.73, p-value = 0.0031). However, we report all model results in the results table of the subsequent section. These regression models consider whether the defense is able to contain receiver i on play j (dwin_i,j_) as a function of the coverage scheme, environment of play, and the receiver being covered. While most of these variables were defined previously, we note that the first specification considers only whether a play occurred in the NFL or in the NCAA (*NFL*_*i*_), whereas the second specification considers the level and season of play (*NFL*14_*i*_, *NFL*15_*i*_). For *doublecover*_*i*,*j*_, the reference group is the set of single-coverage plays. For *NFL*_*i*_, the reference group is the set of plays occurring in the NCAA. Our central research question considers whether doublecover_i,j_ is significantly negative. If so, a necessary condition for the alliance formation puzzle is met. If not, the puzzle could not hold in this setting. Model results are given in Table 3, where all estimated coefficients are in raw form.

**Table 3:** Logistic regression with fixed effects results.

Variable	(1)	(2)	(3)	(4)
Doublecover	0.557***	0.558***	0.709***	0.737***
	(6.326)	(6.332)	(7.72)	(7.99)
NFL	0.067			-0.213
	(1.289)			(-0.76)
NFL_2014		0.061		
		(1.065)		
NFL_2015		0.075		
		(1.262)		
receiver_FEs	--	regressed quietly	--	regressed quietly
Receiver_REs	--	--	regressed quietly	--
_cons	-0.624***	-0.624***	-0.765***	***
N	8,508	8,508	8,508	8,508

Across the three specifications, we find that receivers consistently and significantly enjoy *higher* success rates in single-coverage (e.g., consistently at the α = 0.01 significance level). That is to say, allied defenders enjoy a greater equilibrium probability of containing a given defender than do single defenders. We conclude strong evidence that an alliance formation puzzle does not exist within the American football reception-coverage contest, given strong underlying evidence that the puzzle’s necessary condition is absent. Within an athletic contest that features high marginal payoffs, it appears that capacity constraints prevent an alliance formation puzzle from emerging, as consistent with the theoretical contribution of Konrad and Kovenock [4]. Table 4 shows the marginal effect of double-coverage (as compared to single-coverage) upon the defensive coverage success rate.

**Table 4:** Marginal effect of double-coverage on defensive success likelihood, *c*.*p*.

Model	Marginal Effect of Double-Coverage
1	0.13***
2	0.13***
3	0.16***
4	0.17***

Estimated with all other explanatory variables held at means.

Double-coverage appears to increase defensive secondary success likelihood by between 0.13 and 0.17, *ceteris paribus*. In other words, a secondary defender contributes one additional defensive coverage “win” (containment) for every six to eight plays spent under a double-coverage scheme, on average. This effect is both substantial and highly significant. There is strong evidence that double-coverage increases equilibrium defensive success rate.

These empirical results cannot be attributed to differences in the abilities of secondary defenders (ability of the receiver) across schemes or to the level/season of play. Through receiver and setting-of-play fixed effects, we control for receiver ability and setting-of-play across trials. While the primitive data does not include secondary defender fixed effects for reasons discussed previously, we know that single-coverage is undertaken disproportionately by high-ability cornerbacks, where cornerbacks are coverage specialists. Double-coverage is undertaken disproportionately by a non-“lockdown” cornerback paired with a safety, where safeties are defensive generalists (see, e.g., [22]). For the most part, safeties are not as able *in coverage defense* as cornerbacks. If safeties were more capable in coverage, then a large number of safeties would be moved to the cornerback position to specialize in coverage. We utilize NFL Combine and Draft data in S3 Appendix to consider the differences in cornerback and safety attributes in greater depth.

In the absence of defender fixed effects, we can utilize this institutional knowledge to conclude that the representative double-coverage defender is not a more capable *coverage* defender than the representative single-coverage defender. As such, we conclude that the double-coverage success premium does not derive from (missing) defender fixed effects. If anything, it appears that the double-coverage success premium occurs in spite of lesser raw, per capita coverage ability in a representative double-coverage play. In the empirical results, we also find that setting-of-play is not significantly related to defensive coverage success. While NFL secondary defenses were more successful in sample than their NCAA counterparts, the difference was not significant at any reasonable level. However, receiver fixed effects demonstrate a great deal of heterogeneity in defensive coverage success across the set of receivers defended.

## V. Conclusion

Herein, we have demonstrated a version of the alliance formation puzzle that aligns with reception-coverage contests in American football. In our theoretical treatment, we find that defenses have a lower equilibrium success rate in preventing the receiver from “getting open” under double-coverage than under single-coverage in the absence of (sufficiently-binding) capacity constraints. We also find that this success rate paradox is a necessary condition for the alliance formation puzzle. In the empirical treatment, however, we find that equilibrium level of defensive success rises significantly (p-value < .01) and substantially (marginal effect of between 13 and 17 percentage points) when moving from single-coverage to double-coverage, *ceteris paribus*. There is strong evidence that the necessary condition for the alliance formation puzzle does not hold in this setting. We conclude that sufficiently-binding physiological and training-based capacity constraints eliminate the alliance formation puzzle in this setting, as was shown theoretically by Konrad and Kovenock [4]. This empirical result suggests that other contest settings that regularly feature alliance (e.g., liquidity-constrained conflict) may not be puzzling.

This empirical result may generalize to additional settings of importance. For example, high stakes resource conflicts in underdeveloped regions may feature liquidity constraints that explain alliance formation (e.g., among rebel factions). Moreover, firms may lack the infrastructure or liquidity to devote an optimal number of resources to a particularly valuable research effort. In such a case, joint research and development agreements need not (necessarily) worry about a welfare-eroding collective action problem. Future research may seek to determine empirically whether binding capacity constraints are at play in cases of armed conflict alliance in which the prize is objectively-valued (e.g., cases of resource conflict) and, if so, whether the alliance formation puzzle is present in such environments.

## Supporting information

S1 Appendix Contest with alliance and endogenous prize division. (DOCX)

S2 Appendix Tables summarizing defensive success rate by receiver. (DOCX)

S3 Appendix Institutional, anatomical, and physiological comparison: Cornerback v Safety[23–26]. (DOCX)
