# Supplementary material for: The alliance formation puzzle in contests with capacity-constraints: A test using American football reception-coverage contest data
Source: PLoS One. 2020 Mar 4;15(3):e0227750. doi: 10.1371/journal.pone.0227750 (PMC7055841; doi:10.1371/journal.pone.0227750)
Supplement: S1 Appendix — (DOCX) [file pone.0227750.s001.docx]

**Appendix I: Contest with Alliance and Endogenous Prize Division**

To demonstrate that the alliance formation puzzle is not specific to the theoretical environment chosen, we consider an alternative setting in Appendix 1. In the alternative setting, the prize of containing a receiver is allocated endogenously between the two receivers (i.e., is not subject to even, exogenous division).

In Stage 1, defenders ally with one another in an attempt to contain the receiver under double coverage. In the event that the receiver gains separation, the receiver wins the contest, and the game terminates after one stage. In the event that the defenders succeed in containing the receiver in the first stage, they then compete with one another in a second stage nested contest to establish better apparent position at the close of the play (where the winner of this second stage contest receives credit for the containment). This second stage nested contest between defenders is often apparent as a jockeying for position and even carries on after the play finishes in many cases (whereby each defender indicates that he in fact stopped the play by drawing an “X” with his arms).

We solve this contest through backward induction. Thus, we begin with Stage 2.

Stage 2:

Cornerback: $\pi_{C,2}=\frac{e_{C,2}}{e_{C,2}+e_{S,2}}V-e_{C,2}$

Safety : $\pi_{S,2}=\frac{e_{S,2}}{e_{C,2}+e_{S,2}}V-e_{S,2}$

Taking first order conditions and solving for the first stage equilibrium as in the main model, we obtain

$$e_{C,2}^{*}=e_{S,2}^{*}=\frac{V}{4}$$

$$\pi_{C,2}^{*}=\pi_{S,2}^{*}=\frac{V}{4}$$

Each defender allocates the same effort in the second stage internal conflict. However, only one defender (the one with the best apparent position relative to the receiver) receives the prize. We note that the second stage welfare is implicit in the first stage payoffs. Hence, the payoffs above represent game payoffs to the defenders.

Stage 1:

Receiver: $\pi_{R,1}=\frac{e_{R,1}}{e_{R,1}+e_{C,1}+e_{S,1}}V-e_{R,1}$

Cornerback Defender: $\pi_{C,1}=\frac{e_{C,1}+e_{S,1}}{e_{R,1}+e_{C,1}+e_{S,1}}\pi_{C,2}^{*}-e_{C,1}^{*}$

Safety Defender: $\pi_{D2,STAGE1}=\frac{e_{S,1}+e_{C,1}}{e_{R,1}+e_{C,1}+e_{S,1}}\pi_{S,2}^{*}-e_{S,1}^{*}$

While the defenders have the same expected prize (from the second stage, we note that the second stage game does not allow them to share the prize equally. Rather, they must compete for a winner take all prize in the second stage. Taking first order conditions and solving for the first stage equilibrium as in the main model, we obtain:

$e_{R,1}^{*}=\frac{4V}{25}$

${(e}_{C,1}+e_{S,1})$^*^$=\frac{V}{25}$

${(\pi}_{C,1}+\pi_{S,1})$^*^$=\frac{V}{20}+\frac{V}{20}-\frac{V}{25}=\frac{3V}{50}<\frac{2V}{9}$

The alliance formation puzzle holds in an environment in which the defensive coverage prize is divided endogenously and not necessarily evenly at the play level.
