# Supplementary material for: The alliance formation puzzle in contests with capacity-constraints: A test using American football reception-coverage contest data
Source: PLoS One. 2020 Mar 4;15(3):e0227750. doi: 10.1371/journal.pone.0227750 (PMC7055841; doi:10.1371/journal.pone.0227750)
Supplement: S2 Appendix — (DOCX) [file pone.0227750.s002.docx]

**Appendix 2: Tables Summarizing Defensive Success Rate by Receiver**

Table 2.1 demonstrates defensive success rate by receiver for the NCAA in 2014. Tables 2.2 and 2.3 provide this data for the NFL in 2014 and 2015, respectively.

**Appendix Table 2.1: Defensive Success Rate by Receiver, NCAA 2014**

| **Player** | **Mean** | **Std. Dev.** | $\mathbf{n}_{\mathbf{i}}$ |
| --- | --- | --- | --- |
| Aaron Burbridge | 0.457 | 0.500 | 138 |
| Braxton Miller | 0.290 | 0.461 | 31 |
| Charone Miller | 0.452 | 0.500 | 84 |
| Corey Coleman | 0.307 | 0.464 | 75 |
| De’Runnya Wilson | 0.441 | 0.499 | 102 |
| Demarcus Robinson | 0.397 | 0.493 | 58 |
| Josh Doctson | 0.272 | 0.447 | 125 |
| Kenny Lawler | 0.346 | 0.478 | 130 |
| Keyarris Garrett | 0.317 | 0.468 | 104 |
| Laquon Treadwell | 0.254 | 0.437 | 134 |
| Leonte Carroo | 0.362 | 0.483 | 94 |
| Malcolm Mitchell | 0.263 | 0.443 | 80 |
| Michael Thomas (OH St) | 0.311 | 0.465 | 74 |
| Michael Thomas (SMU) | 0.314 | 0.466 | 121 |
| Pharoh Cooper | 0.494 | 0.503 | 77 |
| Rashard Higgins | 0.258 | 0.440 | 93 |
| Roger Lewis | 0.484 | 0.502 | 122 |
| Sterling Shepard | 0.177 | 0.383 | 130 |
| Tajae Sharpe | 0.420 | 0.495 | 169 |
| Tyler Boyd | 0.506 | 0.503 | 79 |
| Will Fuller | 0.374 | 0.486 | 107 |
| **Total** | **0.356** | **0.479** | **2,127** |

**Appendix Table 2.2: Defensive Coverage Success Rate by Receiver, NFL 2014**

| **Player** | **Mean** | **Std. Dev.** | $\mathbf{n}_{\mathbf{i}}$ |
| --- | --- | --- | --- |
| Allen Robinson | 0.328 | 0.471 | 128 |
| Andre Johnson | 0.302 | 0.460 | 182 |
| Antonio Brown | 0.202 | 0.402 | 218 |
| Brandin Cooks | 0.467 | 0.501 | 135 |
| Charles Johnson | 0.359 | 0.482 | 128 |
| Davante Adams | 0.550 | 0.499 | 191 |
| Demaryius Thomas | 0.417 | 0.495 | 163 |
| Dez Bryant | 0.278 | 0.449 | 194 |
| Donte Moncrief | 0.341 | 0.477 | 79 |
| Dwayne Bowe | 0.475 | 0.501 | 122 |
| Jarvis Landry | 0.394 | 0.491 | 104 |
| John Brown | 0.275 | 0.449 | 80 |
| Jordan Matthews | 0.398 | 0.492 | 93 |
| Justin Hunter | 0.689 | 0.465 | 122 |
| Kelvin Benjamin | 0.388 | 0.489 | 165 |
| Kenny Stills | 0.421 | 0.495 | 133 |
| Martavias Bryant | 0.299 | 0.460 | 97 |
| Michael Crabtree | 0.301 | 0.460 | 146 |
| Mike Evans | 0.377 | 0.486 | 162 |
| Mike Wallace | 0.372 | 0.485 | 164 |
| Odell Beckham Jr. | 0.216 | 0.413 | 194 |
| Percy Harvin | 0.448 | 0.500 | 87 |
| Randall Cobb | 0.297 | 0.458 | 145 |
| Sammy Watkins | 0.527 | 0.500 | 220 |
| Stevie Johnson | 0.255 | 0.438 | 98 |
| **Total** | **0.374** | **0.484** | **3,550** |

**Appendix Table 2.3: Defensive Coverage Success Rate by Receiver, NFL 2015**

| **Player** | **Mean** | **Std. Dev.** | $\mathbf{n}_{\mathbf{i}}$ |
| --- | --- | --- | --- |
| Albert Wilson | 0.341 | 0.476 | 138 |
| Allen Robinson | 0.277 | 0.449 | 213 |
| Anquan Boldin | 0.496 | 0.502 | 115 |
| Calvin Johnson | 0.427 | 0.496 | 211 |
| DeSean Jackson | 0.302 | 0.461 | 126 |
| DeVante Parker | 0.573 | 0.496 | 164 |
| Devin Funchess | 0.533 | 0.501 | 105 |
| Donte Moncrief | 0.345 | 0.477 | 168 |
| Dorial Green-Beck | 0.384 | 0.488 | 159 |
| Doug Baldwin | 0.328 | 0.471 | 116 |
| Golden Tate | 0.458 | 0.500 | 142 |
| Jamison Crowder | 0.446 | 0.500 | 101 |
| Kamar Aiken | 0.308 | 0.463 | 133 |
| Marvin Jones | 0.439 | 0.499 | 107 |
| Mike Evans | 0.311 | 0.464 | 180 |
| Sammy Watkins | 0.298 | 0.459 | 171 |
| Torrey Smith | 0.349 | 0.479 | 126 |
| Travis Benjamin | 0.369 | 0.484 | 130 |
| Tyler Lockett | 0.231 | 0.424 | 108 |
| Willie Snead | 0.322 | 0.469 | 118 |
| **Total** | 0.374 | 0.484 | 2,831 |
