# Supplementary material for: The alliance formation puzzle in contests with capacity-constraints: A test using American football reception-coverage contest data
Source: PLoS One. 2020 Mar 4;15(3):e0227750. doi: 10.1371/journal.pone.0227750 (PMC7055841; doi:10.1371/journal.pone.0227750)
Supplement: S3 Appendix — (DOCX) [file pone.0227750.s003.docx]

**Appendix 3: Institutional, Anatomical, and Physiological Comparison: Cornerback v Safety**

Whereas single-coverage is usually undertaken by cornerbacks, double-coverage typically adds a *designated* free (or sometimes strong) safety who covers “over the top” of the receiver or between the receiver and the receiver’s goal line (for a discussion of coverage schemes, see, e.g., [23] or [24]). Cornerbacks specialize in covering receivers, while safeties are defensive generalists. In football, the primary attribute of a strong reception defender is speed according to Gil Brandt, NFL Media Senior Analyst and revolutionary scout [25]. This is followed by agility and jumping ability. According to NFL player census data from *NFL Savant* [26], cornerbacks have the fastest average straight-line speed of any position in the NFL. The average NFL combine 40-yard dash time for a cornerback currently in the NFL is 4.47 seconds. On average, they are faster than the wide receivers they cover (4.49 seconds), than the running backs they sometimes cover (4.53 seconds), than free safeties (4.54 seconds), or than strong safeties (4.55 seconds).

Then, safeties are a bit slower than the receivers they cover. Cornerbacks lead all positions in terms of agility, as determined by average three-cone drill time. Cornerbacks average 6.88 seconds, whereas wide receivers average 6.9 seconds, running backs average 6.99 seconds, strong safeties average 7 seconds, and free safeties average 7.02. Cornerbacks also lead all positions in terms of average broad jump (121.9 inches). They are followed by free safeties (121.11 inches), wide receivers (120.87 inches), strong safeties (120.32 inches), and running backs (118.87). Cornerbacks and safeties are almost indistinguishable in terms of vertical leaping ability, on average, with cornerbacks (36.26 inches) falling between free safeties (36.3 inches) and strong safeties (36.14 inches). These are the three highest-ranked positions in terms of average vertical leap. Importantly, cornerbacks average the strongest average draft position of any NFL player position, whereas the free safety position ranks thirteenth, and the strong safety position ranks fourteenth.

The cornerback position is exceptionally valuable, then, and cornerbacks have both absolute (as determined by NFL Combine census parameter values) and comparative (as determined by designated specialization) advantages over safeties in terms of reception coverage.
